# Disadvantages of photodynamic therapy for polypoidal choroidal vasculopathy

**DOI:** 10.4103/0301-4738.71691

**Published:** 2010

**Authors:** Jay Kumar Chhablani

**Affiliations:** Shiley Eye Center, University of California, San Diego, La Jolla, CA 92093, USA

Dear Editor,

We read the article by Mitamura *et al*.[1] with interest. The article compares the short-term therapeutic effects of intravitreal bevacizumab (IVB) to those of photodynamic therapy (PDT) for polypoidal choroidal vasculopathy (PCV).

We would like to comment about the application of PDT in PCV. PDT is associated with several disadvantages. First, PCV often presents as multiple widely distributed lesions, so it might be difficult to treat all lesions, including multiple polyps and interconnecting vessels, with a single beam of PDT. Treatment of leaking polypoidal dilations only without treating the entire vascular complex can result in persistence or worsening of exudation. Second, it can be difficult to treat nodules in the peripapillary area with a round PDT beam. Third, features commonly associated with PCV such as a large PED or a large submacular hemorrhage are not usually amenable to PDT treatment. Fourth, PCV tends to recur repeatedly so multiple PDT treatments are often necessary, which can increase the risk of long-term choroidal atrophy. Cases of massive subretinal/suprachoroidal hemorrhage have been reported soon after PDT.[2] Even 50% reduced light fluence PDT can produce a retinal pigment epithelial tear in pigment epithelial detachment.[3]

Recently, Kokame *et al*.[4] reported stabilization of vision at 6 months, with monthly intravitreal injection of ranibizumab in PCV. Lai *et al*. reported stabilization of vision and reduction in exudative detachment with IVB but its limited role in regression of polypoidal lesions in indocyanine green angiography (ICGA).[5] Complete regression of polypoidal lesions in ICGA may not be the therapeutic target but the close follow-up is mandatory. Polyps showing “washout phenomenon” on ICGA can be watched. Considering the disadvantages and economic burden associated with PDT, anti-VEGF drugs alone could be the preferred treatment for symptomatic PCV.
